# A Retrospective Analysis of Characteristics and Outcomes of Young ST-Elevation Myocardial Infarction Patients in the Dubai Population

**DOI:** 10.7759/cureus.85565

**Published:** 2025-06-08

**Authors:** Eyas B Nabhan, Haitham Al-Hashimi, Salah Aldeen Roqia, Basel Baaj

**Affiliations:** 1 Graduate Medical Education, Mohammed Bin Rashid University of Medicine and Health Sciences, Dubai, ARE; 2 Cardiology, Rashid Hospital, Dubai, ARE

**Keywords:** cardiovascular risk factors, coronary artery disease, dyslipidemia, myocardial infarction risk stratifications, myocardial infarction, st-elevation myocardial infarction, united arab emirates, young adults

## Abstract

Background

ST-elevation myocardial infarction (STEMI) is a leading cause of morbidity and mortality, with rising incidence among younger individuals. Conventional risk factors, smoking, diabetes, dyslipidemia, and hypertension, contribute significantly to atherosclerosis, but regional data remain limited. In the UAE, STEMI often presents at a younger age than in Western populations, yet demographic, clinical, and outcome data for this group are still underreported.

Methods

This retrospective study included adults ≤45 years admitted with STEMI to major government hospitals in Dubai (2018-2023). Clinical, demographic, and angiographic data were analyzed. Patients were stratified into low-, intermediate-, and high-risk groups based on left ventricular ejection fraction (LVEF), coronary vessel involvement, and STEMI-related complications. Intergroup comparisons used the Fisher-Freeman-Halton exact test and the Kruskal-Wallis test. Associations between risk category and in-hospital outcomes were assessed using Fisher’s exact test (p < 0.05). Regional findings were compared with global data to identify population-specific trends.

Results

A total of 461 patients met the inclusion criteria and were stratified by risk group. The cohort was predominantly male (98%) and South Asian (86.3%), with a median age of 40 years. Most had ≥1 cardiovascular risk factor: dyslipidemia (59.7%), smoking (55.5%), diabetes (47.5%), hypertension (33%), and a family history of premature CAD (9.5%). The left anterior descending artery was the most common culprit artery (60.3%), and single-vessel disease predominated (48.8%). Most had an uncomplicated hospital course (84.8%), while 15.2% experienced complications-mainly arrhythmias (10%), followed by death, cardiogenic shock, and others. Risk group distribution was as follows: low (70.1%), intermediate (27.5%), and high (2.4%). Significant associations were found between risk categories and dyslipidemia (p = 0.026), age (p = 0.010), eGFR (p = 0.0125), and factors used in stratification: LVEF and vessel count (p < 0.001). Higher risk groups were linked to an increase in in-hospital complications and death (p < 0.001).

Conclusions

Young adults presenting with STEMI in Dubai tend to exhibit shared demographic and anatomical characteristics. Increasing age and dyslipidemia are associated with a higher likelihood of in-hospital complications and adverse outcomes. While some findings, such as the associations with LVEF, eGFR, and extent of coronary disease, aligned with global data, traditional risk factors like diabetes and hypertension showed no significant associations, possibly reflecting population-specific patterns. Although not directly tested, public health strategies targeting smoking cessation and early detection of lipid disorders may help mitigate the burden of premature STEMI. Further research into genetic and metabolic predispositions is warranted to refine risk assessment and improve preventive efforts in this population.

## Introduction

ST-elevation myocardial infarction (STEMI) results from acute total occlusion of one or more of the coronary arteries that supply the myocardium [1], and the pathophysiology of the majority of STEMI cases involves atherosclerotic plaque rupture at the site of occlusion, which is classified as type 1 myocardial infarction [2]. Studies showed the main independent risk factors for atherosclerosis, hence the STEMI, as follows: older age, smoking, diabetes mellitus, dyslipidemia, systemic hypertension, obesity, and male gender [3]. The fundamental of acute myocardial infarction (AMI) treatment is urgent revascularization, preferably through primary percutaneous coronary intervention (PCI) [4]. Although AMI prevalence increases with age, the incidence in younger individuals (<45 years) has risen significantly in recent years [5].

Ischemic heart disease, which includes STEMI, is the leading cause of death worldwide and in the Middle East region [6]. Despite improved survival rates of patients with AMI in recent years, those patients can develop medical, particularly heart failure, and non-medical complications following the event, adversely affecting physical, psychological, financial, and social aspects of these patients’ lives [7] and imposing a significant economic burden on healthcare systems [8].

Possibly due to the rising prevalence of conventional cardiovascular risk factors [9], it is estimated that approximately a third of AMI cases occur in young adults [10]. Patients admitted with AMI in the Middle East tend to be seven to ten years younger than their Western counterparts [11]. This earlier onset is partly attributed to a higher prevalence of diabetes, obesity, and metabolic syndrome in Middle Eastern populations [11].

Given that nearly half of the UAE's population falls within the 15 to 35-year-old age group, according to reports by the UAE Federal Competitiveness and Statistics Centre, this younger bracket is emphasized to reflect the country’s youthful demographic profile. Although our study defines 'young' patients as those under 45 years of age, understanding the characteristics and risk factors within this broader context remains critical. Such insights will support the development of population-specific treatment protocols, surveillance strategies, and preventative measures targeting this predominantly productive segment of society that may experience long-term consequences.

Data on the clinical and demographic characteristics, outcomes, and risk factor prevalence of young STEMI patients in the Gulf region, particularly the UAE, remains limited.

This study aimed to analyze the demographic, clinical, and risk factor profiles of young STEMI patients presenting to major governmental hospitals in Dubai between 2018 and 2023, institutions chosen for their representative patient populations and centralized healthcare data.

Additionally, it applied a simplified point-based risk stratification method to explore associations with adverse outcomes and to contextualize these findings through comparison with published regional studies and international registries, facilitating comparison with global data.

## Materials and methods

This retrospective cohort observational study reviewed electronic medical records of patients who presented to the Emergency departments of Dubai's governmental hospitals, including Rashid and Dubai Hospitals, between January 2018 and January 2023. Additionally, it included patients referred from Hatta Hospital, a non-PCI facility, to undergo primary PCI. These patients were managed in accordance with international STEMI transfer protocols, with standardized timelines to ensure prompt reperfusion therapy comparable to those admitted directly to PCI centers.

Eligible participants were young adults aged 18 to 45 years at the time of admission who were diagnosed with STEMI, underwent coronary angiography, with or without subsequent angioplasty, and had both echocardiographic and coronary angiographic evaluations documented during the index hospitalization. Exclusion criteria included patients outside the specified age range, those who did not undergo coronary angiography, individuals whose primary diagnosis was determined to be other than STEMI upon further clinical evaluation, and patients with incomplete or missing data critical to the study's analysis. Ethical approval for this study was obtained from Dubai Health and Dubai Scientific Research Ethics Committee (DSREC), Dubai Health Authority (reference number: DSREC-SR-03/2024_08).

Data regarding demographic characteristics, clinical details, laboratory results, and treatment approaches were collected. This included factors such as age, sex, ethnicity, body mass index (BMI), and smoking history, defined as current or past tobacco use, as well as a family history of premature coronary artery disease (CAD), defined as the occurrence of CAD in first relative degree younger than 45 years for men and 55 years for women. The clinical data included the BMI, as well as the presence of the following cardiovascular disease risk factors: diabetes mellitus, dyslipidemia, systemic hypertension, and estimated glomerular filtration rate (eGFR).

Cardiovascular risk factors, including diabetes mellitus, hypertension, dyslipidemia, smoking, and family history of premature CAD, were identified based on documented history in the medical records, clinical findings during admission, medication use, or abnormal laboratory values using hospital reference cutoffs.

The Investigations and treatment details included angiographic data, focusing on the culprit lesion responsible for the patient's presentation, as well as the number of vessels with obstructive lesions defined as CAD with ≥50% stenosis, whether it was one, two, or three or more.

Additionally, the study collected specific clinical data during the hospital stay, including left ventricular ejection fraction (LVEF) as assessed by echocardiography, and the in-hospital short-term outcome, which was categorized as either uncomplicated or complicated by one or more complications. These complications included STEMI-related outcomes such as death, cardiogenic shock, mechanical complications, and clinically significant arrhythmias. All complications were recorded regardless of whether multiple events occurred in the same patient.

Arrhythmias were defined as any documented tachyarrhythmia or bradyarrhythmia, including both supraventricular and ventricular types.

Cardiogenic shock was defined as persistent hypotension (systolic blood pressure <90 mmHg for ≥30 minutes or requiring inotropic/vasopressor support), accompanied by clinical signs of hypoperfusion (e.g., cold extremities, altered mental status, low urine output), in the absence of other causes such as hypovolemia or sepsis.

The outpatient follow-up status (i.e., whether the patient attended a follow-up visit or not) was recorded. Although patients were typically scheduled for follow-up approximately one month after discharge, follow-up was considered present regardless of the exact timing. No clinical data from these visits were included in the outcome analysis.

The clinical definition of myocardial infarction, according to the Fourth Universal Definition of Myocardial Infarction [12], specifies the occurrence of acute myocardial injury detected by abnormal cardiac biomarkers in the setting of evidence of acute myocardial ischemia. Acute myocardial injury is defined as the detection of elevated cardiac troponin values above the 99th percentile upper reference limit, along with a rise and/or fall in the levels of these focused cardiac troponin values.

In clinical practice at Dubai Health hospitals, high-sensitivity cardiac troponin T is the primary biomarker used to assess myocardial injury. Troponin levels are typically obtained upon patient presentation and repeated within 3-6 hours, as part of the diagnostic workup.

However, for patients presenting with STEMI, the diagnosis is established primarily based on electrocardiographic (ECG) criteria, in accordance with international guidelines. Specifically, STEMI is defined by the presence of new ST-segment elevation at the J point in at least two contiguous leads, measuring ≥ 2 mm in men or ≥ 1.5 mm in women in leads V2-V3, and/or ≥ 1 mm in another contiguous chest or limb leads. Additionally, a new or presumed new left bundle branch block is considered a STEMI equivalent [12]. In such emergency scenarios, the decision to activate the catheterization laboratory is made based on ECG findings alone, without waiting for troponin results, to avoid treatment delays. Nevertheless, troponin testing is still performed and documented as part of the formal diagnostic and prognostic evaluation for myocardial infarction.

The laboratory analyses obtained at baseline during the hospital admission were done within the medical laboratory department of Dubai's healthcare facilities. The echocardiography and coronary angiography reports were retrieved from the patient's electronic medical records and initially reviewed and reported by the non-invasive and catheterization laboratory teams, respectively.

To identify patient characteristics associated with an increased risk of a complicated clinical course, patients were categorized into three risk groups using a simplified risk score developed by the authors. This score was informed by established risk assessment models such as TIMI [13], GRACE [14], and SYNTAX [15] and adapted to fit the clinical variables consistently available in our retrospective dataset. The goal was to classify STEMI patients into three risk categories, considering three key clinical variables: LVEF%, the number of major coronary vessels with obstructive lesions, and the occurrence of specific STEMI-related Complications (arrhythmias, cardiogenic shock). These three variables were selected based on clinical relevance, predictive value in established literature, and completeness of data in our cohort. Other traditional risk score components were excluded due to inconsistent documentation or limited availability in retrospective records.

Each variable was assigned a score of 1, 2, or 3 points based on the severity, and the cumulative score determined the final risk category for each patient (Table 1). Patients with a total score of 3-4 points were classified as "Low Risk", those with 5-6 points were "Intermediate Risk", and those with 7-9 points were categorized as "High Risk".

**Table 1:** Risk assessment tool and points assigned according to multiple variables LVEF, Left ventricular ejection fraction; STEMI, ST-elevation myocardial infarction. Table Credit: Created by the authors based on multiple established risk scores [13–15].

Risk Factor	Low (1 point)	Moderate (2 points)	High (3 points)
LVEF %	>50% (normal)	35–50% (mildly reduced)	<35% (severely reduced)
Number of obstructed vessels	1-vessel disease	2-vessel disease	3-vessel or left main
STEMI complications	None	Arrhythmias	Cardiogenic shock

Statistical analyses

Categorical variables were summarized as frequencies and percentages, while continuous variables were summarized as medians with interquartile ranges (IQRs) due to non-normal distribution, as confirmed by the Shapiro-Wilk test. For comparisons between the three risk groups, the Fisher-Freeman-Halton exact test was used for categorical variables, and the Kruskal-Wallis test for continuous variables, considering the non-normal distribution of the data.

The Fisher-Freeman-Halton exact test was also used to assess associations between risk categories and adverse in-hospital STEMI outcomes (including all types of post event complications, and mortality).

Variables were selected based on clinical relevance and statistical significance in univariate analyses. The results were reported with 95% confidence intervals (CIs) and p-values.

Missing data composed less than 5% of the dataset and appeared to be missing completely at random (MCAR), as verified through visual inspection of the data patterns. As no formal statistical test (e.g., Little’s MCAR test) was conducted, this assumption is based on the absence of observable patterns of systematic missingness. Pairwise deletion was applied to minimize the loss of statistical power while retaining all available data. Given the minimal level of missingness and the MCAR assumption, more complex methods such as multiple imputation were not deemed necessary. A two-tailed p-value <0.05 was considered statistically significant. Effect sizes were calculated for key comparisons to complement p-values. For categorical outcomes, odds ratios with approximate 95% CIs were reported. For continuous variables, median differences across risk groups were calculated to highlight clinically meaningful trends.

No a priori power calculation was performed due to the retrospective design of the study. However, the final sample size of 461 patients was considered sufficient to detect clinically meaningful differences, based on effect sizes reported in similar observational studies.

Since only a small number of key comparisons were made and the analysis was exploratory in nature, formal corrections for multiple testing were not applied. However, we acknowledge the potential for Type I error, and results should be interpreted with caution. All statistical analyses were done using IBM SPSS Statistics for Windows, Version 25 (Released 2017; IBM Corp., Armonk, New York, United States) and Python (SciPy, StatsModels).

## Results

Of the 513 eligible subjects, we excluded 52 subjects who had had either another diagnosis than STEMI (n=28) or missing data (n=24), leaving 461 patients included in the final analysis.

Overall, the study cohort had a median age of 40 years, with 453 (98%) being male. A total of 437 patients (94.8%) had at least one documented cardiovascular risk factor, while 24 patients (5.2%) had none. Among the individual risk factors, dyslipidemia (275, 59.7%), smoking (256, 55.5%), and diabetes mellitus (219, 47.5%) were the most common.

The study population was predominantly composed of individuals of South Asian descent (primarily from India, Pakistan, and Bangladesh), 398 (86.3%), followed by those of Middle Eastern or North African background, 56 (12.15%). Patients from White/Caucasian, 3 (0.7%), Black, 3 (0.7%), and Native American, 1 (0.2%), ethnic groups were underrepresented in the cohort.

The patients were categorized into three risk groups based on the analysis of key clinical variables, including LVEF%, the number of coronary arteries having obstructive lesions, and the occurrence of specific STEMI-related complications (Arrhythmias, cardiogenic shock). The low-risk group consisted of 323 (70.1%) patients, the intermediate-risk group had 127 (27.5%) patients, and the high-risk group included 11 (2.4%) patients.

The baseline characteristics and clinical outcomes were compared across the different risk groups (Table 2).

**Table 2:** Comparison of baseline characteristics according to risk groups Continuous variables are expressed as median [interquartile range] and compared using the Kruskal-Wallis test (H = test statistic). Categorical variables are presented as numbers (percentages) and compared using the Fisher-Freeman-Halton Exact test (FFH), which does not produce a numeric test statistic. Effect sizes are reported for key variables: odds ratios (OR) for categorical outcomes and median differences for continuous variables. ORs for complication rates are shown relative to the low-risk group. BMI, body mass index; eGFR, estimated glomerular filtration rate; FHx OF Premature CAD, Family history of premature coronary artery disease; * statistically significant, p ≤ 0.05, CI 95%

Risk level	All N=461	Low N=323 (70.1%)	Intermediate N=127 (27.5%)	High N=11 (2.4%)	p-Value	Test Statistic	Effect Size
Age (years)	40 (35-42)	39 (34-42)	40 (38-42)	41 (38-42.5)	0.010	H = 9.28	
BMI (kg/m^2^)	26.21 (23.8-29.39)	26.14 (23.88-29.37)	26.58 (23.67-29.40)	24.82 (22.2-27.27)	0.610	H = 0.99	
Gender					0.052	FFH	
Male	453 (98.26%)	316 (97.8%)	127 (100.0%)	10 (90.9%)			
Female	8 (1.7%)	7 (2.2%)	0 (0%)	1 (9.1%)			
Diabetes	219 (47.51%)	147 (45.5%)	64 (50.4%)	8 (72.7%)	0.072	FFH	
Hypertension	152 (33%)	101 (31.3%)	49 (38.6%)	2 (18.2%)	0.251	FFH	
Dyslipidemia	275 (59.7%)	182 (56.3%)	88 (69.3%)	5 (45.5%)	0.026	FFH	
FHx OF Premature CAD	44 (9.5%)	33 (10.2%)	10 (7.9%)	1 (9.1%)	0.752	FFH	
Smoker	256 (55.5%)	180 (55.7%)	69 (54.3%)	7 (63.6%)	0.103	FFH	
Number of affected vessels					<0.001	FFH	
1-vessel	225 (48.8%)	213 (65.9%)	12 (9.4%)	0 (0.0%)			
2-vessel	129 (28%)	96 (29.7%)	28 (22.0%)	5 (45.5%)			
3-vessel or more	107 (23.2%)	14 (4.3%)	87 (68.5%)	6 (54.5%)			
Ethnicity					0.371	FFH	
Middle Eastern/North African	56 (12.15%)	36 (11.1%)	20 (15.7%)	0			
South Asian	398 (86.3%)	283 (87.6%)	104 (81.9%)	11 (100.0%)			
White/Caucasian	3 (0.7%)	2 (0.6%)	1 (0.8%)	0			
Black	3 (0.7%)	1 (0.3%)	2 (1.6%)	0			
Native American	1 (0.2%)	1 (0.3%)	0 (0.0%)	0 (0.0%)			
EF%	40% (35-50%)	45% (35-50%)	35% (30-45%)	25% (17.5-30%)	<0.001	H = 55.21	Median diff: Low–Intermediate = 10%
eGFR (mL/min)	110.7 (93.8-116.7)	111.0 (95-116.8)	110.7 (94.4-117.2)	93.8 (77-103.35)	0.0125	H = 8.76	Median diff: Low–High ≈ 17.2 mL/min
Death/Complications	70 (15.2%)	28 (8.7%)	31 (24.4%)	11 (100%)	<0.001	FFH	OR (Intermediate vs Low) ≈ 3.4 OR (High vs Low) = undefined (100%)

**Figure 1:**
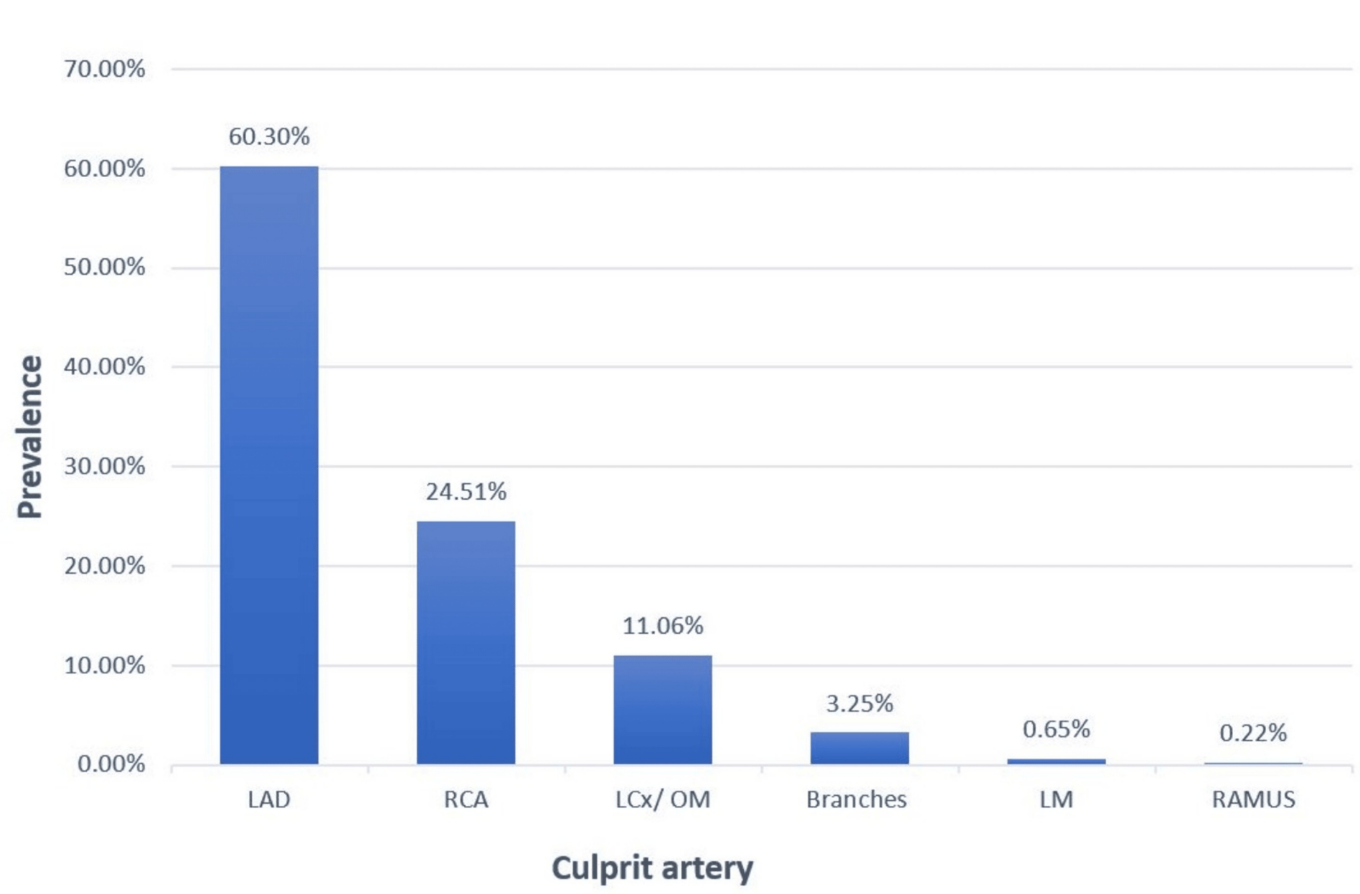
Prevalence of culprit arteries in the study population Branches include PDA: Posterior descending artery, PLV: Posterior left ventricular artery, D: Diagonal artery. LAD, left anterior descending artery; RCA, right coronary artery; LCx/ OM, left circumflex artery/obtuse marginal artery; LMCA, left main coronary artery.

**Figure 2:**
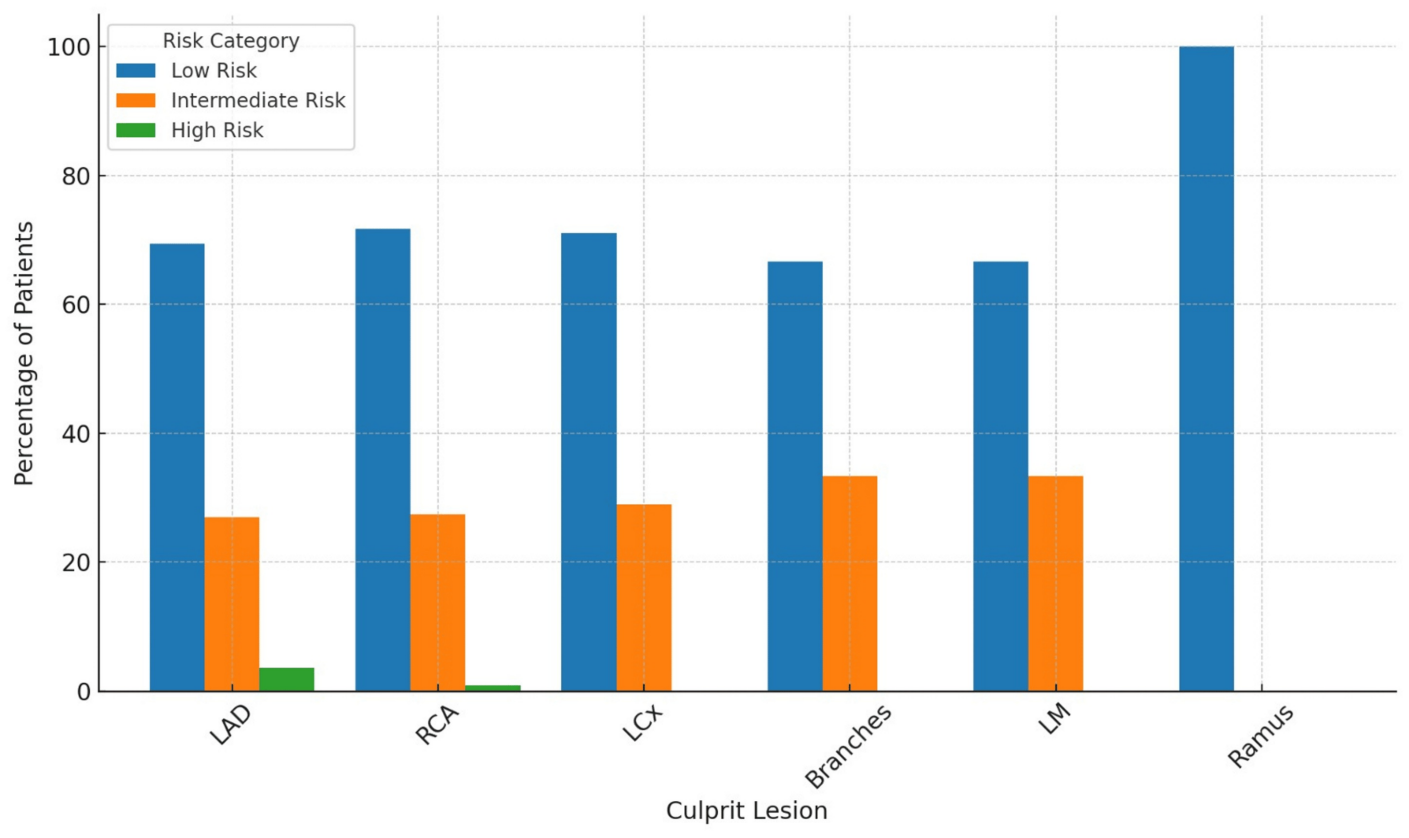
Prevalence of culprit lesions according to each risk group Branches include PDA: Posterior descending artery, PLV: Posterior left ventricular artery, D: Diagonal artery. A p-value of 0.9483 revealed a non-significant association between the risk category and the culprit artery. p-values were calculated using the Fisher-Freeman-Halton exact test (FFH) LAD, left anterior descending artery; RCA, right coronary artery; LCx/ OM, left circumflex artery/obtuse marginal artery; LMCA, left main coronary artery.

The data revealed that the majority of culprit lesions were located in the left anterior descending artery (LAD), 278 (60.3%), while the remaining lesions were distributed as depicted in Figure 1, and in relation to risk groups, Figure 2.

The majority of patients had single-vessel disease, 225 (48.8%), followed by double-vessel disease, 129 (28%), and triple-vessel disease, 107 (23.2%). When it comes to follow-up, most of the patients did not attend scheduled outpatient follow-up appointments after hospital discharge, with only 129 (28%) of the study cohort having at least one post-discharge outpatient visit. The study population predominantly comprised expatriate residents, 443 (96.1%), with UAE nationals constituting only a small fraction, 18 (3.9%) of the cohort.

**Figure 3:**
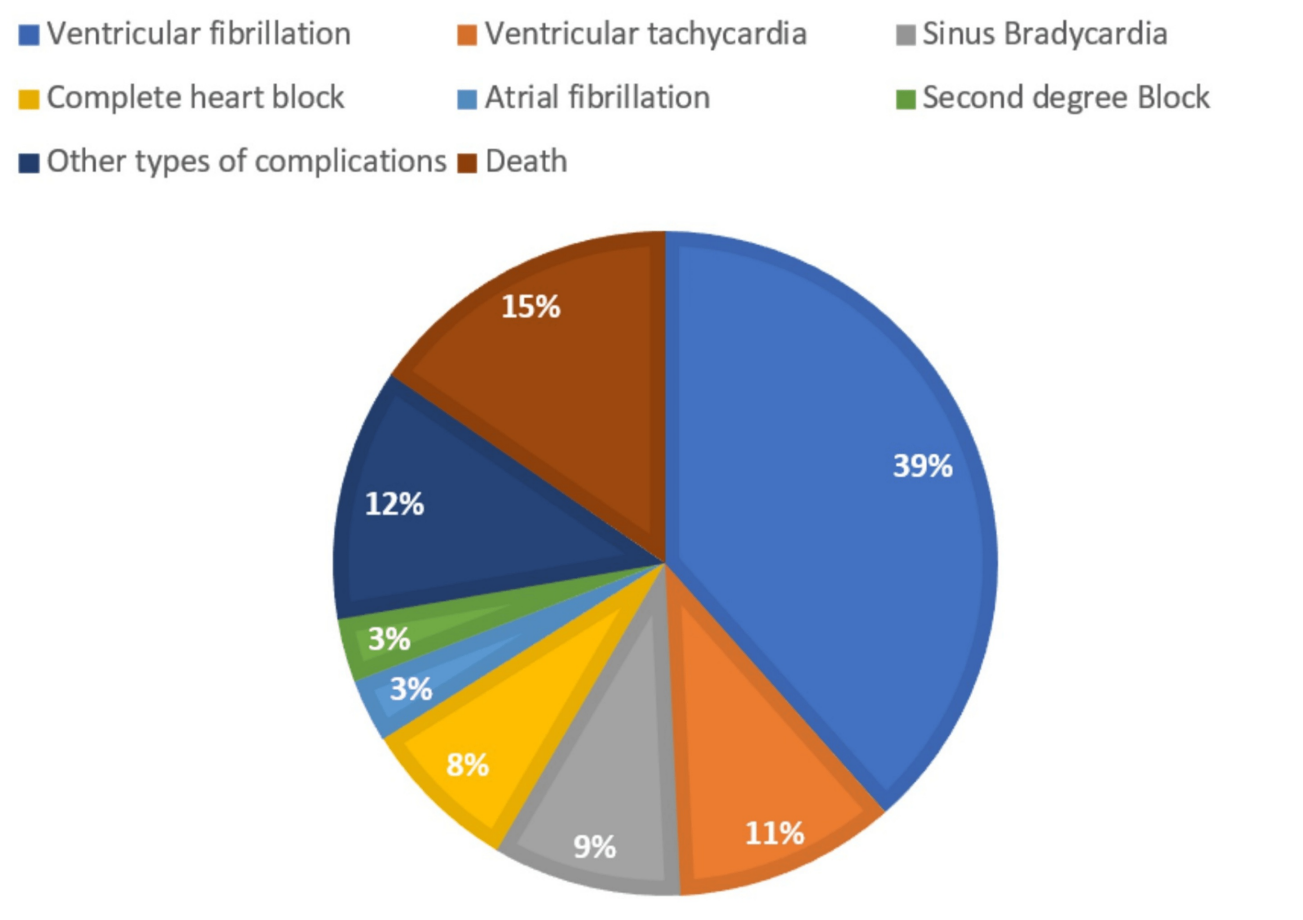
Specific type of complications

Of the total sample, 391 (84.8%) subjects experienced an uncomplicated hospital course during the in-hospital observation period, while 70 (15.2%) had a complicated course with one or more of the following: arrhythmias (46 cases, 10%), death (11 cases, 2.38%), cardiogenic shock (five cases, 1.1%), and a group of infrequent but clinically relevant complications categorized as 'Other Non-STEMI Related Complications' (eight cases, 1.7%). This latter category included rare events such as post-myocardial infarction pericarditis (two cases), left ventricular mural thrombus (four cases), and gastrointestinal bleeding following PCI (one case). These events, while not directly classified as classic STEMI-related complications, were included to provide a comprehensive overview of in-hospital morbidity. Notably, complication categories were not mutually exclusive, and some patients experienced more than one complication. The complications were further subdivided and specified and are demonstrated in Figure 3.

In general, the study population exhibited a decreased LVEF, with a median value of 40% (IQR 35-42%); on the other hand, eGFR was generally preserved with a median value of 110.7 (IQR 93.8-116.7) mL/min/1.73 m^2^.

The study examined the association between different risk categories (low, intermediate, and high) and various clinical and demographic factors using appropriate statistical analyses. The results revealed a significant relationship between risk category and both dyslipidemia (p = 0.026) and age (p = 0.010). Effect size estimates for these variables, including group-wise distributions and median differences, are reported in Table 2.

In contrast, no statistically significant associations were found between risk category and diabetes mellitus, systemic hypertension, family history of premature coronary artery disease, smoking, BMI, or ethnicity. Additionally, there was no significant relationship between the risk category and different types of culprit vessels (p = 0.9483).

Furthermore, the risk category was also significantly associated with LVEF% and eGFR (p < 0.001 and p = 0.0125, respectively). Effect size analysis showed a stepwise decline in median LVEF from 45% in the low-risk group to 25% in the high-risk group and a median eGFR decrease of approximately 17 mL/min between the low- and high-risk groups (see Table 2). However, it is important to note that these parameters were measured after the event during the hospital stay, reflecting post-event cardiac and renal function rather than pre-existing risk factors.

The number of affected coronary vessels was significantly associated with risk groups (p < 0.001). However, it is noteworthy that both the number of affected vessels and LVEF% are already part of the risk stratification criteria, and thus their association is expected.

## Discussion

This study investigated the clinical and demographic characteristics of young STEMI patients who presented to a governmental hospital in Dubai, an underrepresented population in the literature, and an important element of the community who may suffer morbidity and mortality that not only affects the patient and his family but also puts a massive adverse burden on society. In addition, while prior research has largely focused on describing this population, our study aimed to address the gap in outcome-based risk stratification, a topic noted as insufficiently explored in younger patients [16].

We introduced a simplified, clinically informed risk stratification approach based on established markers (e.g., LVEF%, number of affected vessels, and presence of STEMI-related complications). This tool was internally validated by demonstrating statistically significant associations between the assigned risk categories and in-hospital outcomes, suggesting that it may capture meaningful differences in severity. While not a formal predictive model, this stratification framework provides a foundation for future prospective validation and refinement in similar populations.

The cutoff age definition for young individuals vs. old ones has always been a matter of debate in the literature reviewing similar populations as ours, but 45 years old was set according to the United Nations criterion [17]. The findings align with existing regional studies, such as Dugani et al. [18], which report earlier STEMI onset in the Middle East and North Africa (MENA) populations compared to Western equivalents [11,18]. Consistent with prior findings [19-22], this study confirms that young STEMI patients are predominantly male, smokers, overweight, and of South Asian descent, an ethnic group shown to have higher rates and earlier onset of atherosclerotic cardiovascular disease. The overwhelming male predominance (98%) limits the generalizability of these findings to female patients, highlighting the need for further research in this population. Given that South Asians comprise over 70% of Dubai’s expatriate population [23], the findings underscore a pressing public health concern.

Angiographically, findings were also comparable with the literature [24], which showed that the majority of young STEMI patients had the LAD as the predominant culprit lesion, with single-vessel coronary artery obstructive disease being the most common finding. Although no significant association was found between risk category and the specific culprit vessel in our analysis, this may indicate that the anatomical location of the lesion has less prognostic impact in a relatively young, low-comorbidity population. 

Furthermore, Gupta et al. [25] found better short-term outcomes (up to 30 days post-event) in young STEMI patients, which is consistent with our findings. These outcomes may be partly explained by the lower burden of comorbidities, timely presentation, and fewer contraindications to intervention in this younger demographic, factors that have been suggested in prior literature, although not directly assessed in our cohort.

To enhance the clinical interpretation of the cohort and address the limited availability of simplified risk stratification tools specifically designed for young STEMI patients [16], we categorized the study population into three risk groups based on key clinical variables. This approach aimed to facilitate a better understanding of disease patterns, anticipate the in-hospital course, and inform early preventive strategies tailored to this younger demographic. As there are many patient variables and multiple established risk scores that incorporate known risk predictors, a simple stratification system was created using the most relevant elements from these validated tools, adapted to the available data. The validity of this stratification approach was supported by statistically significant associations between the risk groups and key adverse in-hospital outcomes, including complications and mortality (p < 0.001, Fisher-Freeman-Halton Exact test), suggesting that the defined categories meaningfully captured escalating clinical risk. This confirms the internal coherence of the stratification system and supports its exploratory utility for identifying higher-risk patients in this demographic.

Applying the new stratification method, the findings imply significant associations between risk stratification categories and factors such as dyslipidemia, increasing age, lower LVEF, lower eGFR, and the number of affected epicardial coronary vessels. A progressive pattern across risk groups was noted in several significant variables, particularly in the extent of coronary artery disease and post-infarction left ventricular dysfunction [26]. However, no formal trend test was conducted, and these observations should be interpreted cautiously. Notably, dyslipidemia, while statistically significant, did not follow a clear gradient across groups.

Unlike Liang et al. [27], this study did not find significant associations between risk categories and traditional risk factors such as diabetes, hypertension, family history of coronary artery disease, or smoking. While these factors are clinically relevant and widely recognized as contributors to cardiovascular risk, their lack of association in our cohort may reflect the homogeneity of exposure in this relatively young population, limited sample size, or the short-term in-hospital outcome focus. Furthermore, chronic conditions in younger adults may be underdiagnosed or in earlier stages of progression, making their impact on short-term STEMI outcomes less pronounced. These findings should therefore be interpreted with caution and do not diminish the broader relevance of these risk factors in cardiovascular disease prevention.

The strong association between the number of affected coronary vessels with obstructive lesions, LVEF%, and risk category reinforces prior evidence that these factors are clinically relevant in risk stratification and prognosis; furthermore, the number of affected vessels reflects how extensive atherosclerosis is. Therefore, these findings reinforce known clinical patterns but should not be interpreted as independent associations in this analysis.

It is important to highlight that both LVEF and eGFR were assessed after the index STEMI event should be interpreted as markers of post-infarct physiological impact rather than baseline predictive variables. In particular, reduced LVEF is a well-known consequence of myocardial stunning following acute infarction. Although eGFR differed significantly across risk groups, values remained within normal ranges, and the clinical significance of this finding may be limited. Furthermore, the small number of patients in the high-risk group may have influenced these associations, which should be interpreted with appropriate caution.

The lack of a stepwise progression for dyslipidemia across risk categories may reflect the influence of unmeasured factors such as genetic predisposition, medication adherence, or variations in lipid subfractions not assessed in this study. For example, elevated levels of lipoprotein(a) (Lp(a)), which were not measured here, have been associated with premature atherosclerosis and may contribute to cardiovascular risk independent of traditional lipid markers [28].

Additionally, dyslipidemia may drive chronic endothelial dysfunction and low-grade vascular inflammation, mechanisms that do not always align with acute risk stratification models focused on short-term complications [29]. These interpretations are speculative within the limits of our dataset and suggest the need for future studies including detailed lipid profiles and treatment adherence metrics.

While not directly assessed in this study, these findings support the rationale for early and tailored preventive interventions in young adults, particularly those at risk of premature atherosclerosis or with ethnic predispositions to cardiovascular events. As a public health implication, population-level screening programs, especially those targeting lifestyle modification such as smoking cessation, routine lipid profile monitoring, and aggressive management of modifiable risk factors, may help reduce the burden of early-onset STEMI in the MENA population. These strategies warrant further investigation in prospective studies.

The simplified risk stratification approach employed in the analysis may serve as a valuable clinical tool for early recognition of high-risk patients, enabling more aggressive management strategies and close in-hospital observation, in addition to emphasizing outpatient follow-up.

As outpatient follow-up data was collected only descriptively and not included in the analysis, the high rate of non-attendance (72%) did not affect the reported in-hospital outcomes. However, this limits any interpretation related to post-discharge care or long-term prognosis.

Key strengths of the study lie in extensive clinical and angiographic data, which enable a comprehensive evaluation of young STEMI patients in Dubai and the MENA population. Additionally, it introduces a practical and simplified risk categorization framework specifically designed for this population.

Future research should focus on prospective studies to validate our proposed risk stratification tool and assess its predictive value. In addition, further investigation into the genetic and metabolic factors contributing to dyslipidemia in young STEMI patients could provide a deeper understanding.

Limitations

This study has several limitations. Its retrospective design may introduce selection bias, as inclusion was based on electronic health records labeled with a STEMI diagnosis; patients who were misclassified or not accurately coded may have been inadvertently excluded, and the absence of post-discharge follow-up data limits the ability to assess the impact of risk stratification on longer-term clinical outcomes beyond the in-hospital course.

Furthermore, the study did not address elements such as genetic factors and detailed lipid profile variations, which may have influenced the observed findings regarding dyslipidemia. Finally, additional cardiovascular risk factors, such as physical inactivity, mental health conditions, exposure to acute life stressors, occupational strain, and chronic systemic inflammation, were not included in the analysis.

The small proportion of patients in the high-risk group (2.4%) may limit the statistical power to detect significant differences and reduce the generalizability of subgroup-specific findings. However, this distribution reflects the underlying characteristics of the studied population, predominantly young STEMI patients, who are less likely to present with severe complications. As such, the risk group distribution observed may mirror real-world clinical patterns in similar demographics.

While two complications (arrhythmias and cardiogenic shock) were included as input variables based on a previously validated risk score, the composite outcome in this study comprised a broader range of in-hospital complications. This approach limits the potential for circular reasoning, as the classification and outcome definitions are not fully overlapping. Nonetheless, this methodological consideration is acknowledged as a potential source of bias.

The study's ethnically homogeneous cohort, largely composed of South Asian expatriates, reflects local demographics but may reduce external validity to more diverse populations.

While ethnicity was central in defining the cohort’s composition, it was not significantly associated with the risk stratification categories. This suggests that within this relatively uniform population, other clinical factors, rather than ethnicity, were more influential in determining in-hospital outcomes.

Lastly, only univariate analyses were performed in this study. A multivariate model was not applied due to the limited number of complication events and the small sample size in the high-risk group, in order to avoid model overfitting. As a result, findings should be interpreted as exploratory and hypothesis-generating rather than confirmatory.

## Conclusions

Young adults presenting with STEMI in Dubai tend to share common clinical characteristics, including demographic and anatomical patterns. Increased age and dyslipidemia were associated with a higher likelihood of in-hospital adverse outcomes, including death and complications. These outcomes were directly observed during the hospitalization period and assessed using a simplified risk stratification method. While not directly tested in this study, general population screening initiatives, such as those aimed at smoking cessation, early lipid profile monitoring, and aggressive management of modifiable risk factors, may help reduce the burden of premature STEMI. The proposed stratification system, if validated in larger cohorts, may serve as a practical tool to assist clinicians in early triage and tailored monitoring of young STEMI patients. Future studies should evaluate these strategies in prospective settings and explore the potential role of genetic predisposition and biochemical markers to improve risk assessment.
